# Use of 16S rRNA Gene for Identification of a Broad Range of Clinically Relevant Bacterial Pathogens

**DOI:** 10.1371/journal.pone.0117617

**Published:** 2015-02-06

**Authors:** Ramya Srinivasan, Ulas Karaoz, Marina Volegova, Joanna MacKichan, Midori Kato-Maeda, Steve Miller, Rohan Nadarajan, Eoin L. Brodie, Susan V. Lynch

**Affiliations:** 1 University of California San Francisco, Department of Medicine, Gastroenterology Division, 513 Parnassus Ave, San Francisco, CA 94143–0538, United States of America; 2 Lawrence Berkeley National Laboratory, Earth Sciences Division, 1 Cyclotron Rd., MS70A-3317, Berkeley, CA 94720, United States of America; 3 University of California San Francisco, Clinical Microbiology Laboratory, 185 Berry Street, Suite 290, San Francisco, CA 94107, United States of America; 4 University of California, Berkeley, CA 94720, United States of America; 5 San Francisco General Hospital, Department of Medicine, Bldg 100, San Francisco, CA 94110, United States of America; 6 School of Biological Sciences, Victoria University of Wellington, 34 Kenepuru Drive, Porirua, Wellington, New Zealand; Charité, Campus Benjamin Franklin, GERMANY

## Abstract

According to World Health Organization statistics of 2011, infectious diseases remain in the top five causes of mortality worldwide. However, despite sophisticated research tools for microbial detection, rapid and accurate molecular diagnostics for identification of infection in humans have not been extensively adopted. Time-consuming culture-based methods remain to the forefront of clinical microbial detection. The 16S rRNA gene, a molecular marker for identification of bacterial species, is ubiquitous to members of this domain and, thanks to ever-expanding databases of sequence information, a useful tool for bacterial identification. In this study, we assembled an extensive repository of clinical isolates (n = 617), representing 30 medically important pathogenic species and originally identified using traditional culture-based or non-16S molecular methods. This strain repository was used to systematically evaluate the ability of 16S rRNA for species level identification. To enable the most accurate species level classification based on the paucity of sequence data accumulated in public databases, we built a Naïve Bayes classifier representing a diverse set of high-quality sequences from medically important bacterial organisms. We show that for species identification, a model-based approach is superior to an alignment based method. Overall, between 16S gene based and clinical identities, our study shows a genus-level concordance rate of 96% and a species-level concordance rate of 87.5%. We point to multiple cases of probable clinical misidentification with traditional culture based identification across a wide range of gram-negative rods and gram-positive cocci as well as common gram-negative cocci.

## Introduction

Currently, one of the major challenges for clinical practice and public health surveillance is rapid and accurate identification of infectious agents. In the setting of clinical syndromes such as sepsis, even the presence of a pathogen is often unclear since causative agents are cultured in less than 50% of such cases [1], resulting in empirical treatment of a large number of patients. Furthermore, several studies have demonstrated that rapid, appropriate, and adequate antibiotic treatment significantly improves patient outcomes, particularly in the setting of the intensive care unit and that in the absence of such treatment, patient mortality is approximately doubled [2,3]. Rapid and definitive pathogen identification can facilitate appropriate initiation of antibiotic therapy not only in clinical syndromes, such as sepsis, but also in cases of infections potentially caused by multiple pathogens such as in upper respiratory tract infections (URIs). For example, the common presenting clinical symptoms of URIs, cough and coryza, can not discriminate between bacterial and viral agents, but only the former is appropriately treated with antimicrobial administration. These clinical challenges have resulted in inappropriate antibiotic administration and contributed to a large extent to the relatively recent development of pan-resistant microorganisms [4,5].

Emerging infectious diseases such as SARS and reemergence of common bacterial agents exemplified by the *Bordetella pertussis* epidemic in 2010, required intensive efforts over several weeks by public health personnel working with microbiology laboratory personnel to first detect, then contain, and prevent spread of these infectious agents. In addition, hospital-acquired infections from emerging and reemerging pathogens are growing, leading to increased morbidity, mortality, and health care costs on a national level [6,7]. The application of genomic tools to identify etiological agents of acute disease was highlighted with the 2011 *Escherichia coli* outbreak in Hamburg, Germany. Whole genome sequencing of the etiological agent was achieved within a number of weeks of the outbreak, providing information on the super-toxicity of the strain. Clearly, the first step in infectious disease curtailment is rapid and accurate identification of the pathogen(s) involved in the infection. Despite advances in technology, currently, identification and antimicrobial resistance profiling of microbial species by the majority of public health and hospital microbiology laboratories is largely reliant upon culture-based techniques [8,9]. Such approaches are time-consuming requiring at least 16 hours, but frequently substantially more as in the case of fastidious organisms such as *Mycobacterium* and *Legionella* species. Following culture, further biochemical and antimicrobial resistance testing may be performed which adds to the protracted nature of this process. Hence culture-based identification is time intensive and frequently fails to produce relevant data within the critical window of opportunity to permit rapid and appropriate therapeutic decisions to be made.

Molecular testing allows for a large number of pathogens highly specific and sensitive identification from clinical isolates and clinical specimens. The ability of molecular techniques to identify of pathogens directly from clinical samples makes a rapid identification without recourse to culture possible [10–12]. Such approaches are becoming more common for pathogenic species such as methicillin-resistant *Staphylococcus aureus* and *Clostridium difficile* for whom rapid identification is paramount to improving patient outcomes [13]. Over the past several decades, a number of molecular markers that permit identification of specific microbial taxa and their phylogenetic classification [8,14–18] have been identified. Phylogenetic markers include the presence of specific protein coding or structural genes, the combinations of such genes and their variants, insertion and repeat elements. Among these molecular markers, 16S rRNA, an ∼1500 base pair gene coding for a catalytic RNA that is part of the 30S ribosomal subunit, has desirable properties that allowed it to become the most commonly used such marker. Foremost, the functional constancy of this gene assures it is a valid molecular chronometer, which is essential for a precise assessment of phylogenetic relatedness of organisms. It is present in all prokaryotic cells and has conserved and variable sequence regions evolving at very different rates, critical for the concurrent universal amplification and measurement of both close and distant phylogenetic relationships. These characteristics allow the use of 16S rRNA in the assignment of close relationships at the genus [8] and in many cases at the species level [19–21]. Moreover, dedicated 16S databases [22–24] that include near full length sequences for a large number of strains and their taxonomic placements exist. The sequence from an unknown strain can be compared against these sequences. This last point is particularly relevant in an era where DNA sequencing is rapidly becoming a commodity. Tens to thousands of full-length 16S rRNA gene sequences can be generated using capillary sequencing of cloned PCR products while at least two orders of magnitude more short hypervariable regions (250 to 500 bp) can be generated using next-generation sequencing technologies in a cost effective way [25,26]. While relying on non-full length 16S rRNA gene sequence limits the taxonomic resolution and the specific hypervariable region dictates taxonomic coverage [27,28], it is clear that recent advances in sequencing and 16S rRNA gene sequencing protocols [29] will make this molecular marker a more acceptable means for rapid identification.

Several studies evaluated the usefulness of 16S rRNA gene sequencing for clinical microbiology. Historically, slow-growing mycobacteria have been a major group of organisms for which a plethora of 16S studies exist [30,31]. Drancourt et al. compared phenotypic and 16S based identification for a collection of 177 isolates of which 81 were from medical clinical samples [32]. Bosshard et al. evaluated the suitability of 16S rRNA for the identification of clinical strains of aerobic gram-positive rods [33]. Spilker et al. tested 66 cystic fibrosis sputum isolates for the concordance of 16S and phenotypic identification of *Pseudomonas* species [34]. Despite the existence of these studies, a systematic and broad evaluation of 16S rRNA gene for the identification of clinically relevant organisms is lacking. Moreover, even in the existing studies with a limited breadth of organisms, the identification is based on sequence alignment based similarity against databases with very limited diversity (i.e. MicroSeq [35,36], SmartGene [37], RIDOM [38] and 16SpathDB [39,40] among others), which often results in poor classification depth and ambiguous species level identities.

The goal of this study was to compare the accuracy of 16S rRNA gene based identification to that of non-16S based clinical identification for a broad range of clinically relevant bacterial species using a sequence composition based Naïve Bayes classifier trained with a large, diverse set of sequences from clinically relevant species. Toward these aims, we assembled a culture isolate collection of some of the most common hospital-associated bacterial pathogens as well as endemic community-acquired and less common organisms associated with increased disease burden to determine the accuracy of clinical vs. 16S rRNA gene-based identification of these species. The results of our investigation provide insight into the strengths and limitations of molecular identification using 16S rRNA gene for microbiological identification of common bacterial pathogens.

## Materials and Methods

### Clinical isolate repository

617 isolates were collected from diverse sources including blood, urine, respiratory tract secretions, deep tissue samples, and other body sites for this study. The majority of clinical isolates for this study (n = 510) were acquired from collections maintained at the Clinical Microbiology Laboratory at University of California, San Francisco over a two-year period from 2010–2011. Overall, the isolates represented the most common bacterial pathogens (with the exception of two *Neisseria lactamica* isolates) cultured by the UCSF clinical microbiology lab as well as some less common species associated with severe disease burden such as *Stenotrophomonas maltophilia* and *Burkholderia cepacia* complex. Clinical isolates of *Burkholderia cepacia* complex (BCC) (n = 30) were obtained from the strain collections of the Burkholderia cepacia Research Laboratory and Repository (University of Michigan). Clinical isolates of *Bordetella pertussis* (n = 20) were acquired from California Public Health Department, Microbial Diseases Laboratory (Richmond, CA). For *Neisseria meningiditis*. (n = 28) and *Mycobacterium tuberculosis* (n = 29), considered Biological Safety Level 3 organisms, DNA from clinical isolates was obtained from the Institute of Environmental Science and Research (Wellington, New Zealand) and from collections maintained at MTB Research Laboratory (UCSF, CA), respectively. For each of the clinical identities represented in the repository, Table 1 summarizes the clinical identification method, the number of isolates, and the source of the isolate.

**Table 1:** Clinical identity, identification technique, and source of all the clinical isolates used in this study.

Clinical Identity	Identification Technique	Number of Isolates	Source
*Acinetobacter baumannii*	culture-based	20	Clinical Microbiology Laboratory-UCSF, CA
*Bordetella pertussis*	culture-based	20	Microbial Diseases Laboratory-California Public Health Department, CA
*Burkholderia ambifaria*	*recA*-RFLP; box-PCR fingerprinting	2	*B*. *cepacia* Research Laboratory and Repository-University of Michigan, MI
*Burkholderia cenocepacia*	*recA*-RFLP; box-PCR fingerprinting	6	*B*. *cepacia* Research Laboratory and Repository-University of Michigan, MI
*Burkholderia cepacia*	*recA*-RFLP; box-PCR fingerprinting	7	*B*. *cepacia* Research Laboratory and Repository-University of Michigan, MI
*Burkholderia dolosa*	*recA*-RFLP; box-PCR fingerprinting	3	*B*. *cepacia* Research Laboratory and Repository-University of Michigan, MI
*Burkholderia multivorans*	*recA*-RFLP; box-PCR fingerprinting	3	*B*. *cepacia* Research Laboratory and Repository-University of Michigan, MI
*Burkholderia pyrrocinia*	*recA*-RFLP; box-PCR fingerprinting	3	*B*. *cepacia* Research Laboratory and Repository-University of Michigan, MI
*Burkholderia stabilis*	*recA*-RFLP; box-PCR fingerprinting	2	*B*. *cepacia* Research Laboratory and Repository-University of Michigan, MI
*Burkholderia vietnamiensis*	*recA*-RFLP; box-PCR fingerprinting	4	*B*. *cepacia* Research Laboratory and Repository-University of Michigan, MI
*Citrobacter freundii*	culture-based	20	Clinical Microbiology Laboratory-UCSF, CA
*Enterobacter aerogenes*	culture-based	20	Clinical Microbiology Laboratory-UCSF, CA
*Enterobacter cloacae complex*	culture-based	29	Clinical Microbiology Laboratory-UCSF, CA
*Escherichia coli*	culture-based	30	Clinical Microbiology Laboratory-UCSF, CA
*Enterococcus faecalis*	culture-based	30	Clinical Microbiology Laboratory-UCSF, CA
*Enterococcus faecium*	culture-based	31	Clinical Microbiology Laboratory-UCSF, CA
*Haemophilus influenzae*	culture-based	37	Clinical Microbiology Laboratory-UCSF, CA
*Klebsiella oxytoca*	culture-based	20	Clinical Microbiology Laboratory-UCSF, CA
*Klebsiella pneumoniae*	culture-based	30	Clinical Microbiology Laboratory-UCSF, CA
*Mycobacterium tuberculosis*	nucleic acid hybridization	29	MTB Research Laboratory-UCSF, CA
*Neisseria lactamica*	serotyping; *porA* PCR	2	Clinical Microbiology Laboratory-UCSF, CA
*Neisseria meningitidis*	serotyping; *porA* PCR	28	Institute of Environmental Science and Research-Wellington, New Zealand
*Proteus mirabilis*	culture-based	30	Clinical Microbiology Laboratory-UCSF, CA
*Pseudomonas aeruginosa*	culture-based	30	Clinical Microbiology Laboratory-UCSF, CA
*Serratia marcescens*	culture-based	30	Clinical Microbiology Laboratory-UCSF, CA
*Staphylococcus aureus*	culture-based	30	Clinical Microbiology Laboratory-UCSF, CA
*Staphylococcus epidermidis*	culture-based	30	Clinical Microbiology Laboratory-UCSF, CA
*Stenotrophomonas maltophilia*	culture-based	30	Clinical Microbiology Laboratory-UCSF, CA
*Streptococcus pneumoniae*	culture-based	30	Clinical Microbiology Laboratory-UCSF, CA
*Streptococcus* viridans group	culture-based	31	Clinical Microbiology Laboratory-UCSF, CA

### Culture-based identification of clinical isolates

**Fig 1:**
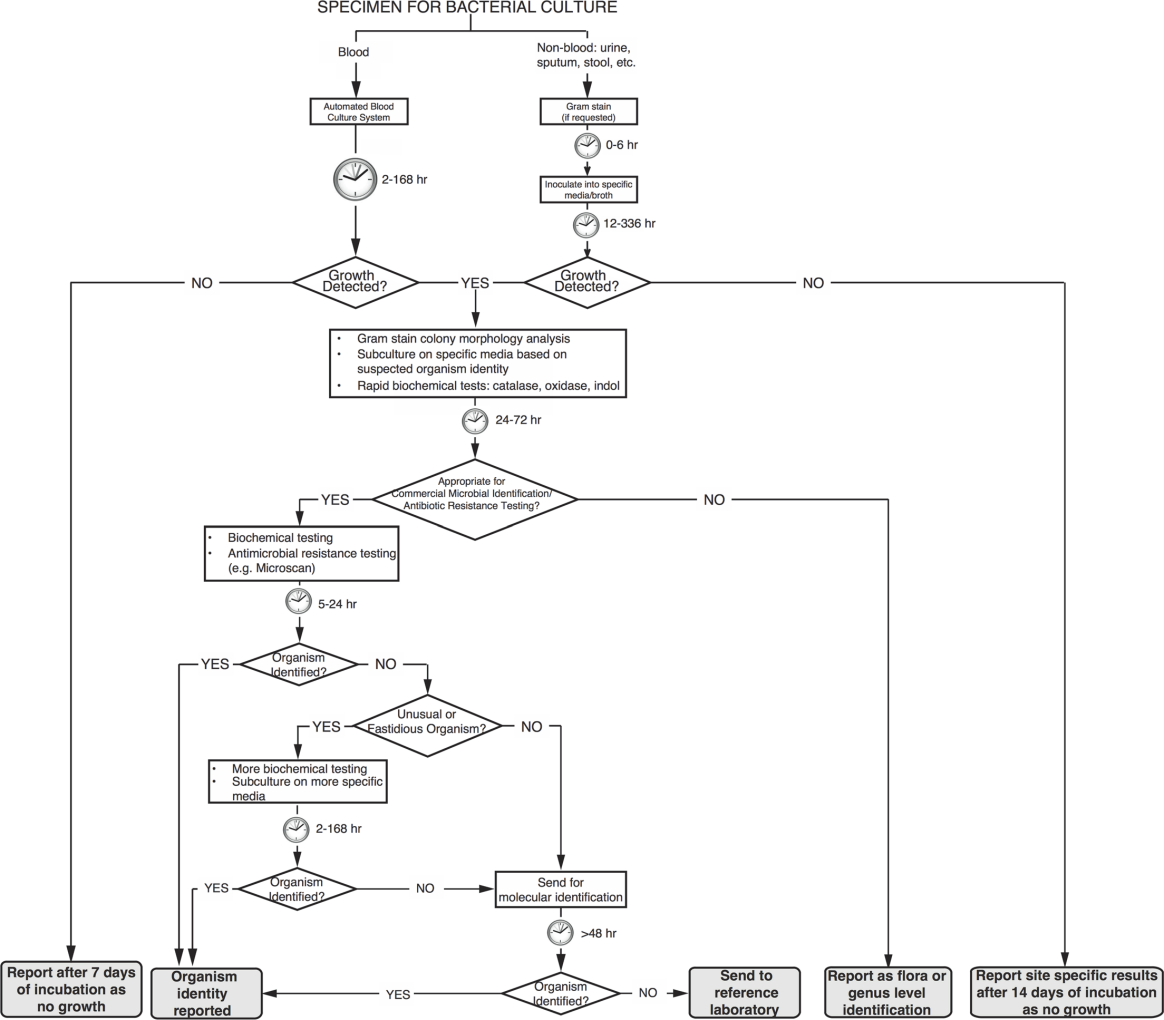
Bacterial identification by clinical microbiology laboratory techniques. Typical temporal workflow of clinical microbiological laboratory to identify microbes from clinical samples based on phenotypic, biochemical, and culture-based techniques.

Isolates obtained from the Clinical Microbiology Laboratory at University of California, San Francisco had undergone culture on relevant selective media, had been further sub-cultured, and had their biochemical profile tested per clinical microbiology laboratory protocols based on current Clinical and Laboratory Standards Institute guidelines to provide a final culture-based identification. Fig. 1 shows the details of the identification protocol and time to results for individual steps.

### Non-16S rRNA-based molecular identification of clinical isolates

*Burkholderia cepacia* complex strains were characterized to the species level by using *recA* RFLP and/or sequence analyses as described [41]. *Mycobacterium tuberculosis* strains were identified using conventional culture techniques with Löwenstein-Jensen media [42] prior to use of AccuProbe (Gen-Probe Incorporated, San Diego, CA), a molecular hybridization protection-based detection assay for *M*. *tuberculosis* identification. *Neisseria spp*. were characterized by serogroup and PorA type utilizing serology and PCR techniques as described elsewhere [43].

### DNA extraction, amplification, and sequencing

The majority of isolates were grown from glycerol stock (generated by the respective labs from a single colony picked from selective media agar plate) on Sheep’s Blood agar plates and incubated for 24–36 hours at 37°C. *Streptococcus spp*. were cultured on Sheep’s Blood agar but incubated at 37°C in a 5% CO_2_ incubator for 24–36 hours. *H*. *influenzae* isolates were first cultured on chocolate agar and incubated in a 5% CO_2_ environment for 24–48 hours. Single colonies of each isolate were sub-cultured in liquid media for DNA extraction. The majority of species were sub-cultured in Luria-Bertoni broth and grown at 37 C and 200 rpm for 24–48 hours, *H*. *influenzae* was sub-cultured on Haemophilus Test Media (HTM) and *Streptococcus spp*. was sub-cultured in custom Trypticase Soy Broth (TSB) in a 5% CO_2_ environment for 24–48 hours. *Bordetella pertussis* isolates were received from the California Public Health Department, in Regan-Lowe agar, initially plated onto Bordet-Gengou agar plates and incubated at 37°C for 6 days; subsequently single colonies were inoculated in Stainer-Scholte broth at 35°C for 24–48 hours [43]. BCC isolates, received from the *Burkholderia cepacia* Research Laboratory and Repository in BBL CultureSwab plus Amies medium with charcoal (BD, NJ), were immediately plated on Typticase Soy agar plates and incubated at 35°C for 48 hours; single colonies from were then inoculated into TSB and grown at 37 C and 220 rpm for 24–48 hours.

A total of 2 ml of liquid culture of each isolate was centrifuged and DNA extracted using a combination of bead-beating (5.5 ms^-1^ for 30s) and the Qiagen AllPrep kit (Qiagen, CA) with the exception of *Neisseria spp*. and *M*. *tuberculosis* strains which had confirmed pathogen-free DNA and provided to the repository from source laboratories as discussed above. Universal 16S rRNA bacterial primers 27F (5’-AGAGTTTGATCCTGGCTCAG-3’) and 1392R (5’-GGTTACCTTGTTACGACTT-3’) were used to amplify this gene using 10 ng of genomic DNA isolated from each strain. PCR products were visualized on a 1% agarose gel stained with ethidium bromide under UV light to confirm the presence of a ∼1,350 bp band. PCR products were purified using EXOSAP-IT (Ambion, CA) prior to bi-directional sequencing using primers 27F and 1392R. Sanger sequences were generated at the UCSF Genomics Core Facility using an ABI PRISM 3730xl DNA Analyzer (Life Technologies Corporation, CA).

### 16S rRNA sequence assembly, quality scoring, and trimming

Each 16S rRNA sequence was base called using Phred (version 0.020425.c). Previous studies have shown that the quality of 16S sequences are essential to accurate phylogenetic placement [44] and taxonomic classification [45]. To obtain the longest feasible high-quality sequences, forward and reverse reads corresponding to each isolate were assembled using Phrap (version 0.990329) with default parameters and the ends of the assembled sequences were trimmed such that any base with a quality score of less than 30 (99.9% base call accuracy) was removed.

### Naïve Bayes classifier for species-level classification of 16S rRNA sequences

The RDP 16S rRNA classifier [46] classifies rRNA sequences down to genus level, which was insufficient for the purposes of our study. To enable the most accurate species level classification based on the paucity of sequence data accumulated in public databases, a Naïve Bayes classifier with a new training set was built.

**Training set.** 16S rRNA sequences with a genus and species name (Isolated_named_strains_16S_aligned.fasta) were downloaded from Greengenes database [23]. This set included 108,413 sequences and was filtered to obtain a set of 35,472 sequences corresponding to medically important bacterial 89 genera listed in the most current edition of *Manual of Clinical Microbiology* [47] (S1 Dataset). All the species (pathogens and commensals) under these genera were included. S2 and S3 Datasets list GenBank accession numbers for the sequences in the training set and the number of sequences for all the genera and species in the training set respectively.

**Training of the Naïve Bayes classifier and genus/species classification of 16S rRNA sequences.** A k-mer based Naïve Bayes classifier was trained using a k-mer length of 8 bp using “classify.seqs” command in Mothur [48]. The assembled 16S rRNA sequences were classified to species level and the bootstrap confidences for the genus and species level classifications were estimated based on 100 iterations.

### Calculation of 16S rRNA percent identities within and between genera/species

**Fig 2:**
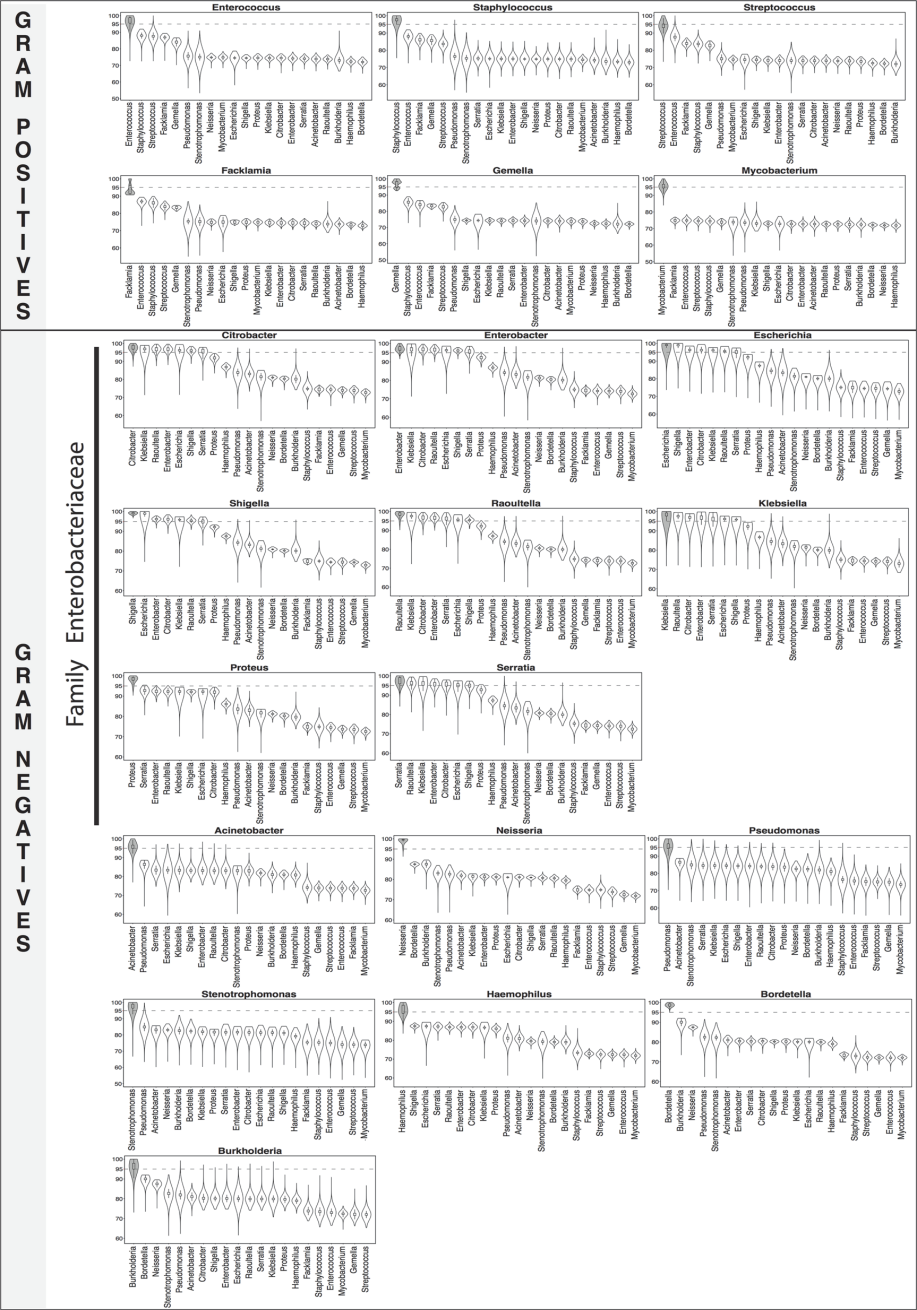
16S rRNA percent identity within and between genera. Distributions (shown as violin plots) of 16S rRNA percent identity (y-axis of each figure) of pairs of training set sequences belonging to the same (gray) and different genera. 95% identity, the traditional genus level cutoff, has been marked for reference. The genus Mycobacterium has been categorized as a gram-positive in the figure. For all of the genera, sequence variability between sequences from the same genera was significantly higher than between those from different genera for all comparisons (Wilcoxon test one-sided p-value<0.0001).

**Fig 3:**
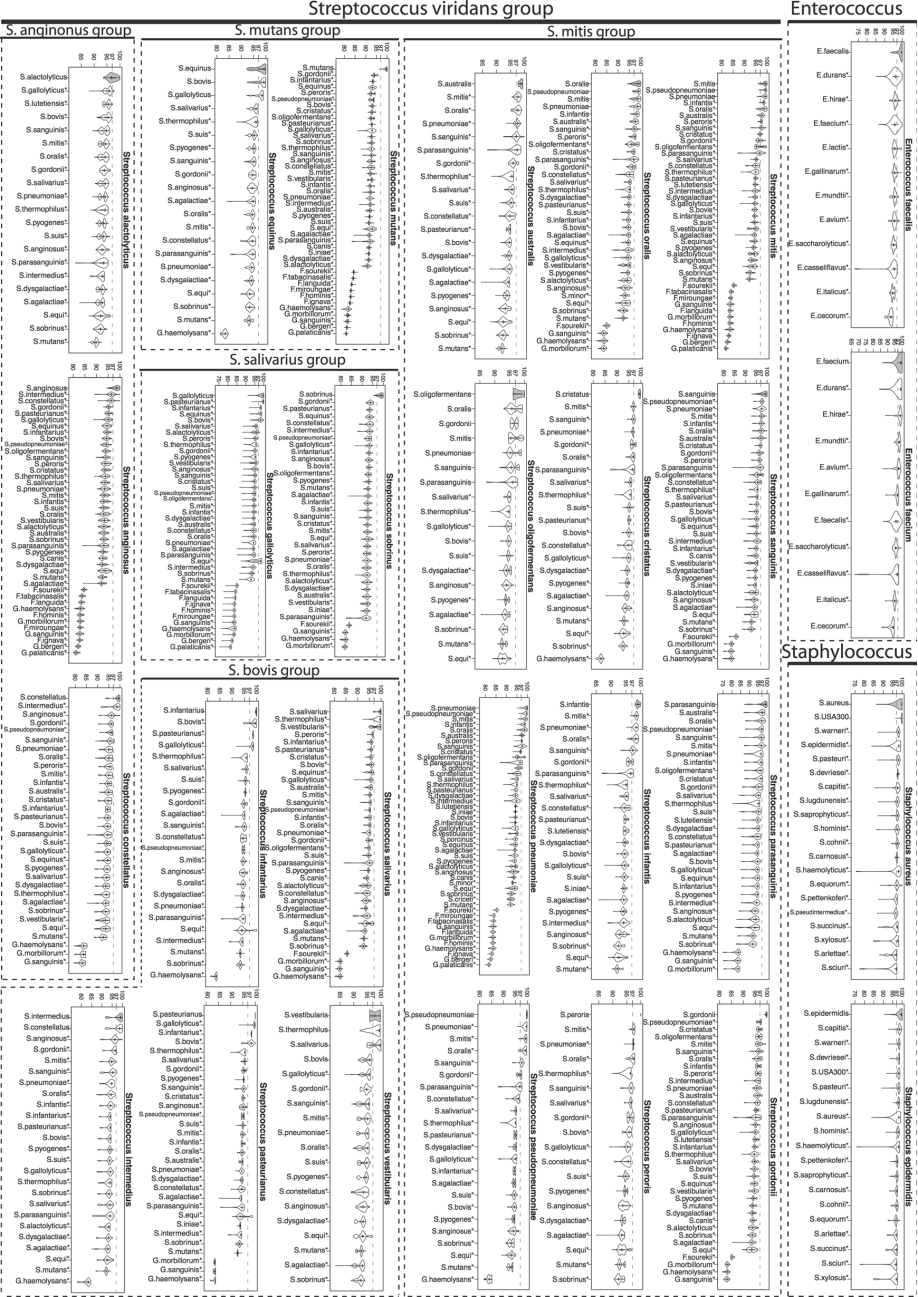
16S rRNA percent identity within and between species (gram-positive bacteria). Distributions (shown as violin plots) of 16S rRNA percent identity (y-axis of each figure) of pairs of training set sequences belonging to the same (gray) and different species for select gram-positive bacteria. 97% identity, the traditional species level cutoff, has been marked for reference. Species for which sequence variability between sequences from the same species was significantly higher than between those from different species are marked with a * (Wilcoxon test one-sided p-value<0.0001).

**Fig 4:**
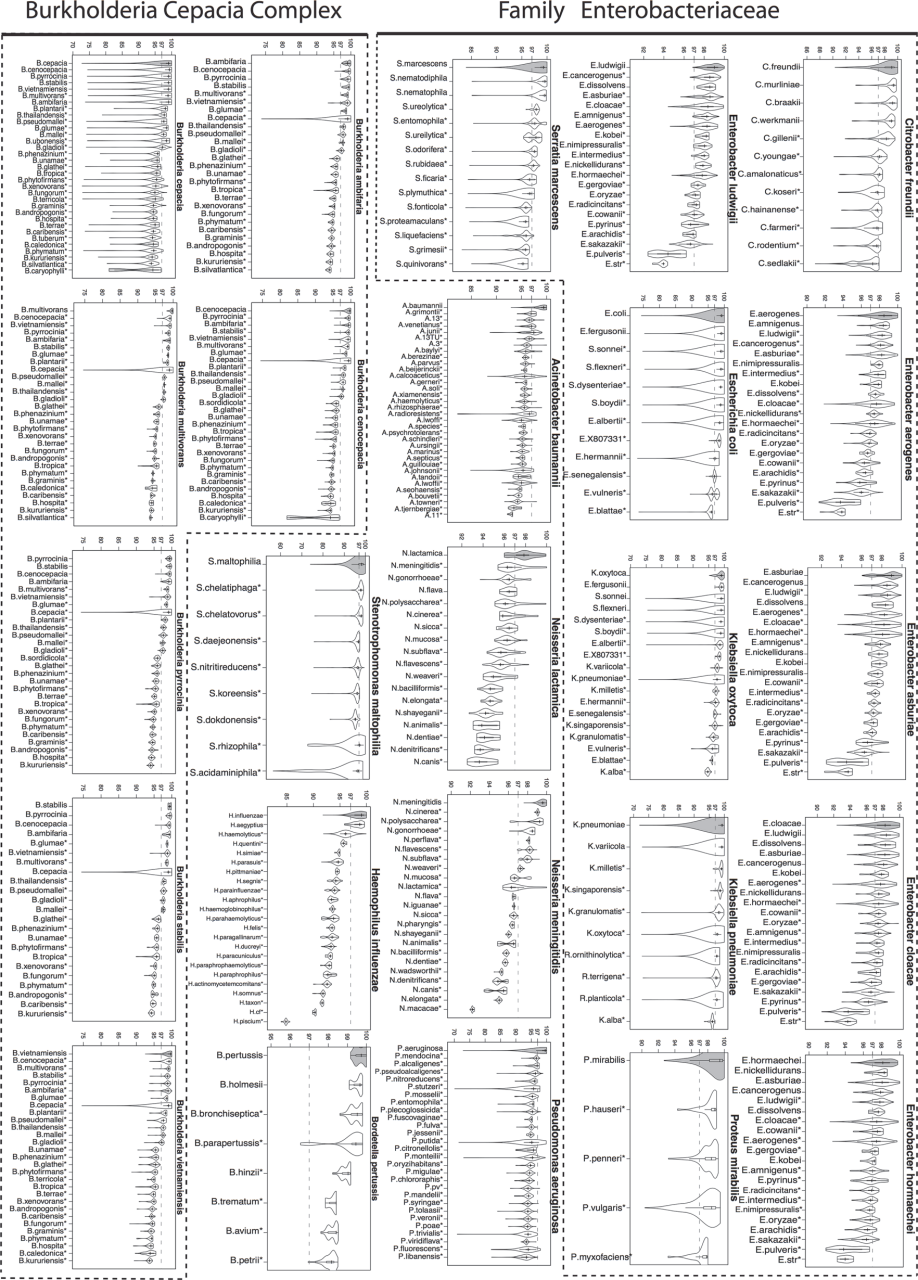
16S rRNA percent identity within and between species (gram-negative bacteria). Distributions (shown as violin plots) of 16S rRNA percent identity (y-axis of each figure) of pairs of training set sequences belonging to the same (gray) and different species for select gram-negative bacteria. 97% identity, the traditional species level cutoff, has been marked for reference. Species for which sequence variability between sequences from the same species was significantly higher than between those from different species are marked with a * (Wilcoxon test one-sided p-value<0.0001).

Training set sequences were aligned to the SILVA Release 119 SEED alignment (available as part of Mothur at http://www.mothur.org/w/images/5/56/Silva.seed_v119.tgz) using “align.seqs” command in Mothur. The SILVA SEED alignment is 50,000 character long. For each pair of sequences, the alignment was parsed out and the positions where the corresponding characters in both sequences were a gap (“-“) or “.” were removed. The percent identity for a pair of sequences was calculated by dividing the number of matches by the total number of the remaining alignment positions. The distributions of within and between genera/species percent identities were obtained pooling sequence pairs from same or different genera/species and visualized as a combination of box plots and kernel density plots using *vioplot* function in R [49] vioplot package (Figs. 2–4). For each genera/species, the within genera/species and between genera/species distributions were compared using the two-sample Wilcoxon test with a one-sided alternative as implemented in *wilcox*.*test* R function.

### Comparisons between clinical and 16S-based identities for heterogeneous group of organisms

Among 19 different clinical identities, two (Enterobacter cloacae complex and *Streptococcus viridans*) corresponded to heterogeneous groups of organisms. To make comparisons between the species-level 16S based identification and the coarser clinical identification feasible, we expanded these two groups of organisms to species level. The currently assigned *E*. *cloacae* complex species include *E. asburiae, E. cloacae, E. hormaechei, E. kobei, E. ludwigii, and E. nimipressuralis [50]. Streptococcus viridans group* consists of the following species [51]: *(1) S*. *mitis group*: *S*. *mitis*, *S*. *sanguinis*, *S*. *parasanguinis*, *S*. *gordonii*, *S*. *oralis*, *S*. *cristatus*, *S*. *infantis*, *S*. *peroris*, *S*. *australis*, *S*. *oligofermentans*, *S*. *pneumoniae*, *S*. *pseudopneumoniae (2) S*. *mutans group*: *S*. *mutans*, *S*. *sobrinus (3) S*. *salivarius group*: *S*. *salivarius*, *S*. *vestibularis*, *S*. *thermophiles (4) S*. *bovis group*: *S*. *equinus*, *S*. *gallolyticus*, *S*. *infantarius*, *S*. *pasteurianus*, *and S*. *alactolyticus (5) S*. *anginosus group*: *S*. *anginosus*, *S*. *constellatus*, *S*. *intermedius*. For these two clinical identities, the 16S-based identity was deemed to be concordant with the clinical identity if it matched any of the species within the respective group.

## Results

A total of 617 clinical isolates had initially been identified using either culture (n = 528 with 19 different clinical identities, Table 1) or non-16S rRNA molecular markers (n = 89 with 11 different clinical identities, Table 1). These initial identities span 17 genera, including a wide range of gram-negative rods and gram-positive cocci as well as common gram-negative cocci. The 16S rRNA gene was amplified and sequenced for each strain yielding a set of high-quality (Q-score > = 30) 16S sequences ranging in length from 751 bp. to 1239 bp. (mean = 1177±41 bp.) (see S4 Dataset).

### Characterization of the Naïve Bayes classifier training set

To assign genus and species identities based on 16S rRNA sequences, we built a 16S rRNA Naïve Bayesian (NB) classifier that can generate species level taxonomic classifications with associated confidence metrics for medically important organisms. The classifier was trained with an extensive set of sequences from Greengenes [23], a curated full-length 16S rRNA gene database (S2 and S3 Datasets).

We characterized this newly created training set through a systematic study of the sequence variability (measured by percent identity (pid)) within and between each genus/species of interest. Though the training set included examples of genera and species matching and not-matching clinical identities (S1 Dataset), we present here a subset of those relevant for comparison of clinical identities with 16S-based identification. Fig. 2 shows the distributions of percent identities of sequences from the same (gray filled violin plots) and different (unfilled violin plots) genera while Figs. 3 and 4 (for gram-positive and gram-negative bacterial species respectively) show the corresponding distributions at the species level. S5 and S6 Datasets summarize basic characteristics of these distributions for genera and species respectively.

At the genus level, the mean pid of sequences belonging to the same genus ranged from 93.84% to 99.15%. Two genera, Facklamia and Streptococcus, had a mean pid < 95% (Facklamia: pid = 93.84+/-3.25%, Streptococcus: pid = 94.46+/-2.33%; S5 Dataset). Since accurate identification of the genus Facklamia is not easy [52] it is possible that Greengenes database is enriched with sequences inaccurately assigned to this genus. Streptococcus is a genus that contains many genetically highly heterogeneous species [51], which is likely to result in many pairs of sequences with low pids. Also within gram-positives, Enterococcus (pid = 96.79+/-2.45%) and Staphylococcus (pid = 97.44+/-2.34%) genera were marked by low-frequency areas in the 72%-95% range (long left-side tails) pointing to the presence of a small yet sizeable number of sequences having low-sequence identities. Within the large family of Enterobacteriaceae, the distributions for Escherichia and Klebsiella genera were significantly less tight than for the other genera. Two species of these two genera (*E*. *coli* and *K*. *pneumoniae*), heavily represented in the training set, are known to have high intraspecific variation between multiple copies of 16S rRNA genes in the genome [53]. For all of the genera, sequence variability between sequences from the same genera was significantly higher than between those from different genera for all comparisons (Wilcoxon test one-sided p-value<0.0001, Fig. 2).

At the species level, the mean pid of sequences belonging to the same species ranged from 97.08% to 100% (S6 Dataset). Comparisons of within and between species pid distributions for 53 different species (Figs. 3 and 4) show that there is significant variability across different species in the predictive power of 16S gene for distinguishing species. For each species, these comparisons show which other species are most likely to be confused for the species in question. For instance, for many species including *S*. *gordonii*, *S*. *mutans*, *S*. *anginonus*, *E*. *faecalis* (Fig. 3), *A*. *baummannii*, *N*. *meningitidis and B*. *multivorans* (Fig. 4), the within species distribution is extremely tight and significantly different than all of the between species distributions (Wilcoxon test one-sided p-value<0.0001) and as a result near full-length 16S gene sequence has considerable power to distinguish these species from other species of the same genus. We observed that particularly species under Enterobacteriaceae family had large spread in their within species distributions (Fig. 4), likely due to a combination of inaccuracies in the Greengenes database and true biological variability, limiting the power of 16S gene for accurate identification of these species. Nevertheless, even for these species, the bulk distribution of within species pid was significantly different than that of between species pid for many other species of the same genus.

### Concordance between 16S rRNA based and clinical identities using Naïve Bayes Classifier and an alignment based approach

Alignment-based sequence similarity methods are commonly used to classify 16S rRNA sequences to the species-level[35–40]. For comparison, we used similarity searches against 16SpathDB (http://147.8.74.24/16SpathDB/main.php), a recent non-proprietary database of medically important bacteria [39,40], and compared the results to the classifications from NB classifier.

For each isolate, its 16S rRNA sequence was used for genus and species identification using the newly constructed NB classifier and the latest version of 16SpathDB. For each of 30 types of initial clinical identities, Table 2 shows the level of concordance between the clinical identity and 16S rRNA based Bayesian and alignment based classifications. Since 16SpathDB identification is based on a "best-hits” approach, in many cases it results in an ambiguous rather than a definite identification leading to multiple identified species including the clinically identified one (Table 2). S4 Dataset lists for each isolate, the initial clinical identification and the genus-species classifications based on the 16S rRNA gene using both methods. For the Bayesian classifier, in addition to the genus and species level classifications, bootstrap estimates of classification confidence are listed for both taxonomic levels. In cases where 16SpathDB gives a multitude of identified species, all the species with the highest percent sequence similarity are listed. At the genus level, 593 (96%) and 580 (94%) of all isolates were concordant with their initial clinical identification using the NB classifier and sequence similarity to the sequences in 16SpathDB 2.0 respectively. At the species level, the rates of concordance with the clinical identities were 87.5% for NB and 80% for 16SpathDB 2.0.

**Table 2:** Concordance rates between clinical and 16S rRNA based identification.

Clinical Identification	16S rRNA Identification					
	Naïve Bayes Classifier		16SpathDB			
	% Concordance		% Concordance		No. isolates with definite identification	
	Genus	Species	Genus	Species	Genus	Species
*Acinetobacter baumannii*	100 (20/20)	85 (17/20)	100 (20/20)	85 (17/20)	20/20	17/17
*Bordetella pertussis*	100 (20/20)	100 (20/20)	100 (20/20)	100 (20/20)	20/20	1/20
*Burkholderia ambifaria*	100 (2/2)	0 (0/2)	100 (2/2)	50 (1/2)	2/2	0/1
*Burkholderia cenocepacia*	100 (6/6)	83.33 (5/6)	100 (6/6)	0 (0/6)	6/6	-
*Burkholderia cepacia*	100 (7/7)	0 (0/7)	100 (7/7)	57.14 (4/7)	7/7	0/4
*Burkholderia dolosa*	100 (3/3)	0 (0/3)	100 (3/3)	100 (3/3)	3/3	3/3
*Burkholderia multivorans*	100 (3/3)	100 (3/3)	100 (3/3)	100 (3/3)	3/3	3/3
*Burkholderia pyrrocinia*	100 (3/3)	0 (0/3)	100 (3/3)	0 (0/3)	3/3	-
*Burkholderia stabilis*	100 (2/2)	0 (0/2)	100 (2/2)	50 (1/2)	2/2	1/1
*Burkholderia vietnamiensis*	100 (4/4)	75 (3/4)	100 (4/4)	0 (0/4)	4/4	-
*Citrobacter freundii*	90 (18/20)	90 (18/20)	85 (17/20)	80 (16/20)	16/17	10/16
*Enterobacter aerogenes*	90 (18/20)	85 (17/20)	90 (18/20)	85 (17/20)	18/18	17/17
*Enterobacter cloacae* complex*	100 (29/29)	96.55 (28/29)	100 (29/29)	6.9 (18/29)	27/29	15/18
*Enterococcus faecalis*	100 (30/30)	96.67 (29/30)	100 (30/30)	96.67 (29/30)	30/30	29/29
*Enterococcus faecium*	100 (31/31)	93.33 (29/31)	100 (31/31)	0 (0/31)	31/31	-
*Escherichia coli*	100 (30/30)	60 (18/30)	100 (30/30)	93.33 (28/30)	30/30	0/28
*Haemophilus influenzae*	94.59 (35/37)	91.89 (34/37)	94.59 (35/37)	89.19 (33/37)	35/35	33/33
*Klebsiella oxytoca*	75 (15/20)	75 (15/20)	30 (6/20)	30 (6/20)	2/6	2/6
*Klebsiella pneumoniae*	93.33 (28/30)	93.33 (28/30)	70 (21/30)	60 (18/30)	21/21	17/18
*Mycobacterium tuberculosis*	96.55 (28/29)	93.1 (27/29)	96.55 (28/29)	96.55 (28/29)	28/28	0/28
*Neisseria lactamica*	100 (2/2)	100 (2/2)	100 (2/2)	100 (2/2)	2/2	2/2
*Neisseria meningitidis*	100 (28/28)	100 (28/28)	100 (28/28)	96.43 (27/28)	28/28	27/27
*Proteus mirabilis*	100 (30/30)	100 (30/30)	100 (30/30)	100 (30/30)	30/30	30/30
*Pseudomonas aeruginosa*	100 (30/30)	96.67 (29/30)	100 (30/30)	96.67 (29/30)	30/30	29/29
*Serratia marcescens*	100 (30/30)	100 (30/30)	100 (30/30)	100 (30/30)	30/30	30/30
*Staphylococcus aureus*	100 (30/30)	100 (30/30)	100 (30/30)	100 (30/30)	30/30	30/30
*Staphylococcus epidermidis*	96.67 (29/30)	76.67 (23/30)	96.67 (29/30)	76.67 (23/30)	29/29	23/23
*Stenotrophomonas maltophilia*	86.67 (26/30)	86.67 (26/30)	100 (30/30)	100 (30/30)	30/30	30/30
*Streptococcus pneumoniae*	100 (30/30)	93.33 (28/30)	100 (30/30)	90 (27/30)	30/30	25/27
*Streptococcus viridans* group*	83.87 (26/31)	77.4 (24/31)	83.87 (26/31)	70.97 (26/31)	26/26	24/26
ALL ISOLATES	96.11(593/617)	87.5(540/617)	94 (580/617)	80 (496/617)	573/580	398/496

16S based genus and species identities were through the use of the Naïve Bayes classifier and an alignment based approach (16SpathDB). For each clinical identity, in addition to the concordance rates, the number of concordant isolates among all isolates is listed in parentheses. For the latter method, the last two columns give the number of isolates with definite identification among the concordant isolates

* For species level comparisons, species for the clinical identifications *Enterobacter cloacae* complex and *Streptococcus viridans* group clinical were: *Enterobacter cloacae* complex *species*: *Enterobacter asburiae*, *Enterobacter cloacae*, *Enterobacter hormaechei*, *Enterobacter kobei*, *Enterobacter ludwigii*, *Enterobacter nimipressuralis*. *Streptococcus viridans* group species: (1) S. mitis group: S. mitis, S. sanguinis, S. parasanguinis, S. gordonii, S. oralis, S. cristatus, S. infantis, S. peroris, S. australis, S. oligofermentans, S. pneumoniae, S. pseudopneumoniae (2) S. mutans group: S. mutans, S. sobrinus (3) S. salivarius group: S. salivarius, S. vestibularis, S. thermophiles (4) S. bovis group: S. equinus, S. gallolyticus, S. infantarius, S. pasteurianus, and S. alactolyticus (5) S. anginosus group: S. anginosus, S. constellatus, S. intermedius.

Bayesian taxonomic classification has a distinct advantage over the simple sequence similarity based method in terms of identification specificity. While the Bayesian classifier predicts a single genus-species identity with the associated confidence levels, sequence similarity based approach of 16SpathDB results in up to six best-hits leading to ambiguous species identification for 107 isolates. Among 496 isolates whose 16SpathDB identification matched with the clinical identity, 398 had definite identification (a single genus-species). Considering the identification specificity at the species level, using the Bayesian classifier, 87.5% of all the isolates had a definite identification in agreement with the clinical identity while the same rate remained at 64.5% using 16SpathDB.

### Clinical identification with culture and non-16S molecular methods and their concordance with 16S rRNA based Bayesian classification

When stratified by whether the initial clinical identification was based on culture or molecular methods, genus-level rates of concordance were roughly similar for either type of clinical identification (Table 3: 98.87% for molecular vs. 96% for culture with NB classifier) while at species-level 16S-based and clinical identification were more concordant for the isolates initially identified using culture-based techniques (Table 3: NB classifier, 90% for culture vs. 76.4% for non-16S molecular with NB classifier). This skew in the concordance rate was primarily due to the isolates from genus *Burkholderia* that were clinically identified using multiphasic diagnostic tests. As these tests are known to be highly accurate for characterization of *Burkholderia* genomovars [54,55], they are unlikely to be clinical misidentifications.

**Table 3:** Concordance rates between 16S rRNA based and clinical identification for isolates clinically identified by culture-based or non-16S based molecular methods.

	Naïve Bayes Classifier		16SpathDB	
	Genus	Species	Genus	Species
Molecular (non-16S based)	98.87 (88/89)	76.4 (68/89)	98.87 (88/89)	77.5 (69/89)
Culture	96 (505/528)	90 (473/528)	93 (492/528)	81 (427/528)

### Confidence of 16S-based species level assignments for the clinical isolates

**Fig 5:**
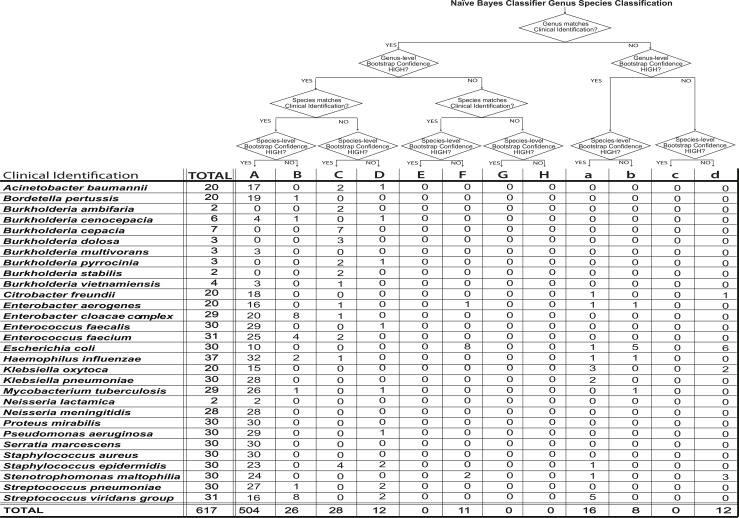
16S rRNA based genus and species level isolate identities with the Naïve Bayes classifier. Each isolate was assigned to one of 12 categories (A-H, a-d) based on the agreement between clinical and 16S rRNA based genus and species classifications and the confidence scores.

A unique feature of the 16S rRNA based Bayesian classifier for the medically important organisms is that in addition to a genus-species classification, it generates bootstrap confidence estimates for both taxonomic levels. Use of a high bootstrap cutoff corresponds to a taxonomic classification with a higher accuracy. We compared genus and species clinical and 16S rRNA-based identities taking into account the classification confidence for the latter (see Fig. 5 and S4 Dataset). The threshold for a high confidence classification was 70 (out of 100 bootstrap samples). These comparisons placed each isolate into one of 12 categories: Categories A-H correspond to cases for which there were genus level concordance between the clinical and 16S rRNA based identities with high (A-D) or low (E-H) bootstrap confidences. Categories a-d corresponds to cases for which 16S rRNA classification was to a different genus with either high (a or b) or low (c or d) bootstrap confidence.

### Clinical isolates with concordant species-level 16S identities with the Bayesian classifier

For 5 out of 30 clinical identities (*S*. *aureus*, *P*. *mirabilis*, *S*. *marcescens*, *N*. *meningitidis*, *N*. *lactamica*), all of the corresponding isolates had their 16S rRNA based genus-species classifications match their clinical identities with high confidence. *B*. *pertussis* clinical identity had all but one of its isolates (BPERT593 with species level confidence = 68, see S4 Dataset) concordant high confidence species level classifications.

### Discordances between clinical and 16S-based identities

The discordances between the clinical and 16S-based identities of the isolates could be due to several factors including (1) insufficient representation of the clinical identities in the training set, (2) phenotypic misidentification, and (3) new taxonomic or phylogenetic placements. The breadth and depth of the NB classifier training set for representation of species corresponding to the clinical identities makes (1) unlikely. The bootstrap confidence score of the best matching taxa generated by the NB classifier gives a level of confidence to the assignment it makes. As such, it is informative on to whether (2) or (3) is more likely to underlie the observed discordances. The assignments with high confidence scores are more likely to be indicative of phenotypic misidentifications while the assignments with low confidence scores are indicative of taxonomic or phylogenetic novelty not represented in the training set.

### Clinical isolates with discordant species-level 16S identities with the Bayesian classifier

For a total of 40 isolates from 18 different clinical identities, NB classifications were to another species under the same genus (categories C and D). Three *A*. *acinetobacter* clinical isolates (AB297, AB468, AB422, see S4 Dataset) classified to *A*. *calcoaceticus-baumannii* (Acb) complex by 16S. The comparison of the distribution of percent identities within (*A*. *acinetobacter*) and between (*A*. *acinetobacter*—*A*. *calcoaceticus*) species suggests that 16S rRNA has considerable power to distinguish these two species (Fig. 4). These are likely to be misidentifications as routine culture based identification of Acb complex with commercial systems such as API and VITEK are often erroneous [56,57].

Within catalase negative gram-positive cocci, *E*. *faecalis*, *E*. *faecium*, *and S*. *pneumoniae*, 5 isolates were placed into categories C or D (EFCA341, EFCI183, EFCI187, SPNE475, SPNE477, see S4 Dataset). Commercial implementations of phenotypic and biochemical tests for species level identification within Enterococci have often been reported to be unreliable and inaccurate [58,59]. The two *E*. *faecium* isolates were identified to be *E*. *durans* and *E*. *faecalis* with high (77 and 100 respectively) bootstrap confidences. One *E*. *faecalis* isolates was classified to be E. faecium with a confidence of 64. Two isolates clinically identified as *S*. *pneumoniae* was re-identified as *S*. *mitis* by 16S. Reliable differentiation between these species is a common challenge in routine clinical microbiology laboratory and misidentifications have been reported using all routinely used identification systems [60–62].

Within genus Burkholderia, with the exception of *B*. *multivorans* and *B*. *vietnamiensis* isolates, the agreement at species-level between the original clinical and 16S-based identifications was poor. For many Burkholderia species, within and between species percent identity distributions were largely overlapping (Fig. 4). The fact that the original clinical identification of all 29 *Burkholderia* isolates was through highly accurate multiple molecular markers confirms that polyphasic approaches for this genus are far superior to a 16S-based identification.

Among the 30 isolates originally identified as *E*. *coli*, 16 were classified as *E*. *coli* and 14 under the polyphyletic genus *Shigella* as *S*. *flexneri* species. Half of the concordant (category F) and all but one of the discordant classifications (categories D and H) had low confidence scores confirming that the variability of 16S rRNA between named *Shigella* and *E*. *coli* isolates is insufficient for a reliable classification (Fig. 4).

Viridans streptococci are fastidious gram-positive cocci with more than 30 recognized species. Species level identification of viridans group streptococci by phenotypic methods is challenging due to natural competence in genus *Streptococcus* [63], which might lead to ambiguous biochemical profiles. NB classifier provided very high confidence species classifications under *Streptococcus* genus for 16 isolates (category A, species level confidence > 90).

### Clinical isolates with discordant genus-level 16S identities from the Bayesian classifier

For five clinical identities within family Enterobacteriaceae (*K*. *oxytoca*, *K*. *pneumoniae*, *E*. *aerogenes*, *C*. *freundii* and *H*. *influenzae*), up to 25% of the isolates per clinical identity were 16S identified to be members of another genus (categories a-d). Significantly, of the 13 isolates in total, eight had very high confidence 16S genus and species classifications (genus and species confidences > 85) and an additional two isolates with a genus level only high confidence classifications. Two *Klebsiella oxytoca* and *one Klebsiella pneumoniae* were classified as *Raoultella ornithinolytica* (isolates KO267 and KO545, see S4 Dataset) and *Raoultella planticola* (isolate KP258, see S4 Dataset) respectively. Misidentification of *R*. *ornithinolytica* and *R*. *planticola* as *K*. *pneumoniae* or *K*. *oxytoca* is common due to the poor performance of routinely used phenotypic identification systems in identifying these species [64] and lack of *R*. *planticola* in the databases of commercialized systems.

NB classifier predicted one isolate which was clinically identified as *C*. *freundii* (isolate CFRE367, S4 Dataset) to be *S*. *marcescens* with genus and species confidence of 100. Clinical misidentification of *S*. *marcescens* with phenotypic tests is common especially with the use of API 20E identification strips (bioMérieux), a widely used commercial system for the identification of gram-negative bacilli [65].

Within clinically identified *S*. *viridans* group isolates, 16S based identification predicted five isolates as species of genera *Facklamia* and *Gemella* with genus and species level bootstrap confidences of 100. These two genera consist of organisms that resemble viridans streptococci and misidentifications of both as *Streptococcus* has been widely reported [66–68].

Four *S*. *maltophilia* isolates were 16S classified into genus Pseudomonas. Among those one was identified to be *P*. *hibiscicola* with high confidence and three to be *P*. *geniculata* with low confidence (isolate SMAL400 under category a and isolates SMAL154, SMAL282, SMAL397 under category d; S4 Dataset). In a clinical microbiology laboratory, the decision to identify many nonfermentative gram-negative rods under genera Pseudomonas and Stenotrophomonas is based on the isolation site. These cases are most probably clinical presumptive misidentification as definitive identification of every nonfermenter isolate is not cost and time efficient and hence not routinely performed.

## Discussion

Accurate identification of bacteria is important for clinical care and public health surveillance to understand the pathobiology of infectious clinical syndromes and better use of specific antibiotic and infection control strategies for patients and populations. Many have questioned whether standard culture-based approaches can correctly distinguish between isolates given that many diverse species can share the same biochemical phenotypes. Indeed, some species not considered “difficult” to identify based on routine culture-based testing were confirmed to be misidentified upon subsequent sequence-based molecular identification [8].

In this study, we used high quality sequences generated from 16S rRNA gene amplicons to compare to culture or non-16S based molecular identification of clinical isolates and ascertain accuracy of routine microbiological identification of a broad range of clinically relevant bacterial species. To our knowledge, our study is unprecedented in terms of its size and breadth. Our repository of clinical isolates for this study included 617 isolates spanning 30 species level clinical identities, with multiple gram-positive and gram-negative cocci, gram-negative rods and the gram-positive rod *M*. *tuberculosis*. While extensive, this set still represents a limited subset of the possible isolates from clinical samples, in particular missing many important pathogens including Group A&B Streptococcus, MOTTs, Listeria, Legionella, Shigella, Yersinia and Campylobacter among others.

For the purposes of this study, to increase species level sensitivity and specificity of 16S based identification, we used a model-based methodology for species level classification of clinical organisms. At species level, our classifier was 87.5% concordant with the clinical identification and outperformed traditional alignment based methodologies. We demonstrated very high confidence genus level identification and good species level identification with exceptions in certain genera such as Stenotrophomonas, Enterobacter, Citrobacter, and Escherichia. The poor species-level identities are likely, as previous described, due to taxonomic ambiguities [8,32] and suggest that this biomarker is not highly discriminatory for such species. Taxonomic debates of ambiguities will, necessarily, continue and likely evolve as more sequence-based bacterial identification data emerges. However, what remains certain is that biomarker bacterial gene sequences remains a valid and non-subjective measure (compared to culture and biochemical assays) of bacterial identity, although as indicated in this study, particular biomarkers may afford poor discriminatory power for particular groups of organisms. Even the current version of Bergey’s Manual of Systematic Bacteriology, one of the most authoritative and widely used manuals by microbiologists to characterize and identify bacteria, utilizes the16S rRNA gene sequence as the template on which to base phylogenetic characterization of prokaryotes including archaea and bacteria [69].

Current tools for 16S based taxonomic classification of clinical isolates use pairwise alignments to a very limited set of sequences from culture collections [35,37,38,40]. In this study, we used a nucleotide composition based model and a curated high-quality set of 16S sequences from a diverse set of medically relevant bacteria. We compared our results to a traditional alignment based method. While the rates of agreement with the initial clinical identification varied between two techniques (Naïve Bayes Classifier and 16SpathDB), these rates aren’t informative as to which method is more accurate overall and within individual genera. Moreover, it is essential to note the fundamentally different philosophy underlying Naïve Bayes classifier compared to a simple comparison with a database of type strains of limited diversity. The diversity of 16S sequences accumulated in databases is reflected in the training set underlying the Naïve Bayes classifier while the type strain depends on a single representative picked relatively arbitrarily which renders it sensitive to “database noise”. An additional benefit of the model based approach is the bootstrap based confidence metric that accompanies the classification. A “best-hit” approach often generates equally valid multiple identities. When the ambiguity of the identification was taken into account, a much larger percentage of the isolates’ 16S identity was in agreement with the clinical identity using our Naïve Bayes classifier compared to 16SpathDB.

The taxonomic breadth and depth (number of sequences for each taxa) of the training set used in building the Naïve Bayes classifier is crucial for maximizing the confidence of taxonomic classifications [21,69]. Among the currently available 16S databases (RDP[22], SILVA [24], and Greengenes [23]), Greengenes is the most diverse. In this study, to build a species-level classifier, we constructed a training set based on Greengenes sequences with available genus and species labels. Though the genera included in the training were limited to those of medical interest, it is important to note that we included all of the species of these genera independent of whether the species had pathogenic association.

The limitations of using molecular identification by 16S rRNA have been described by many and the primary issues have been a lack of high quality sequence generation, reliance on phylogenetic clustering methods and/or sequence alignment scores, databases comprising of misannotated user-submitted sequences, and/or databases with sparse sequence coverage for relevant organisms [8,9,69]. Getting full-length or near full-length 16S sequences is crucial for making confident genus and species level taxonomic placements. While next-generation sequencing technologies have gained popularity for clinical microbiology applications [70], the current sequencing read lengths fall short of reaching the length of the 16S gene, limiting their usefulness for high resolution taxonomic placement. In this study, creation of a well-annotated database of 16S rRNA sequences and use of a naïve Bayes classifier, demonstrate that excellent genus level identification and good species level identification can be achieved. Furthermore, for practical clinical purposes, especially for difficult to culture organisms such as *Burkholderia spp*. and *Mycobacterium tuberculosis*, use of the 16S rRNA gene biomarker offers a more facile and expedient method of identification in the setting of critical patient care needs and population epidemic control efforts. We conclude that routine high confidence identification of bacteria at least to the genus-level can be achieved for a large number of medically important organisms despite poor taxonomic boundaries for certain organisms by utilizing a curated 16S rRNA gene database with a robust naïve Bayes classifier.

## Supporting Information

S1 Dataset Organisms included in the Naïve Bayes Classifier training set. (XLS)

S2 Dataset Naive Bayes Classifier training set sequences and their taxonomic labels. (XLS)

S3 Dataset Number of sequences in the training set for genus and species levels. (XLS)

S4 Dataset 16S based identification for 617 clinical isolates. For each isolate, its original clinical identity is listed together with the 16S rRNA gene sequence and genus/species classifications using Naïve Bayes Classifier and 16SpathDB 2.0.
(XLS)

S5 Dataset Characteristics of within genera percent identity distributions (Fig. 2, gray violin plots). (XLS)

S6 Dataset Characteristics of within species percent identity distributions (Figs. 3 and 4, gray violin plots). (XLS)
